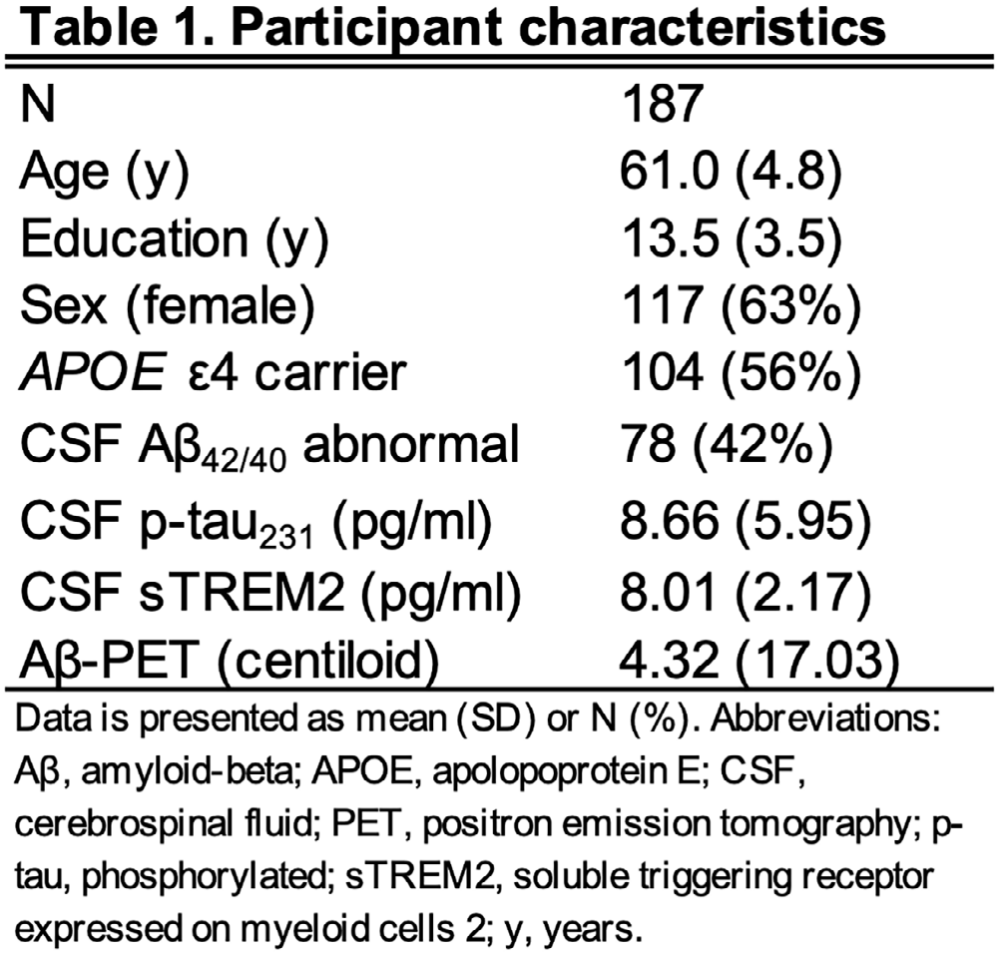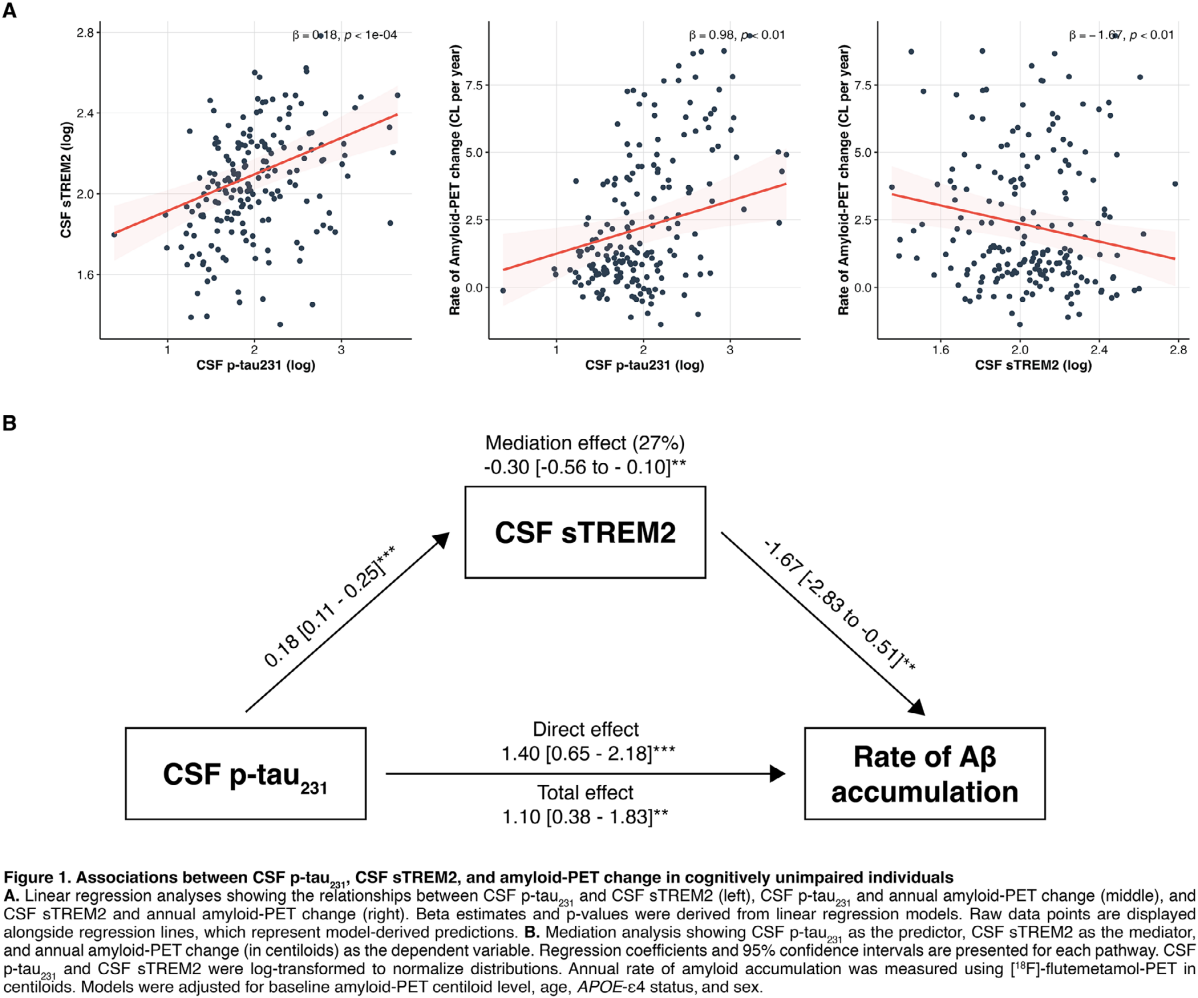# Microglial reactivity attenuates ptau_231_‐related amyloid accumulation in preclinical Alzheimer’s disease

**DOI:** 10.1002/alz70862_110128

**Published:** 2025-12-23

**Authors:** Wiesje Pelkmans, Mahnaz Shekari, Armand González Escalante, Gwendlyn Kollmorgen, Clara Quijano‐Rubio, Kaj Blennow, Henrik Zetterberg, Juan Domingo Gispert, Marc Suárez‐Calvet, Gemma Salvadó

**Affiliations:** ^1^ Barcelonaβeta Brain Research Center (BBRC), Pasqual Maragall Foundation, Barcelona Spain; ^2^ Hospital del Mar Medical Research Institute (IMIM), Barcelona Spain; ^3^ Universitat Pompeu Fabra, Barcelona Spain; ^4^ Hospital del Mar Research Institute (IMIM), Barcelona Spain; ^5^ Roche Diagnostics GmbH, Penzberg Germany; ^6^ Roche Diagnostics International Ltd., Rotkreuz Switzerland; ^7^ Institute of Neuroscience and Physiology, University of Gothenburg, Gothenburg, Mölndal Sweden; ^8^ Paris Brain Institute, ICM, Pitié‐Salpêtrière Hospital, Sorbonne University, Paris France; ^9^ Neurodegenerative Disorder Research Center, Institute on Aging and Brain Disorders, University of Science and Technology of China and First Affiliated Hospital of USTC, Heifei China; ^10^ Clinical Neurochemistry Laboratory, Sahlgrenska University Hospital, Mölndal Sweden; ^11^ Institute of Neuroscience and Physiology, University of Gothenburg, Mölndal Sweden; ^12^ Department of Neurodegenerative Disease, UCL Institute of Neurology, London UK; ^13^ Wisconsin Alzheimer’s Disease Research Center, University of Wisconsin School of Medicine and Public Health, University of Wisconsin‐Madison, Madison, WI USA; ^14^ Clinical Neurochemistry Laboratory, Sahlgrenska University Hospital, Gothenburg Sweden; ^15^ Hong Kong Center for Neurodegenerative Diseases, Clear Water Bay, Hong Kong China; ^16^ Spanish National Center for Cardiovascular Research (CNIC), Madrid Spain; ^17^ Centro de Investigación Biomédica en Red de Bioingeniería, Biomateriales y Nanomedicina (CIBER‐BBN), Madrid Spain; ^18^ AstraZeneca, Barcelona Spain; ^19^ Centro de Investigación Biomédica en Red de Fragilidad y Envejecimiento Saludable (CIBERFES), Madrid Spain; ^20^ Servei de Neurologia, Hospital del Mar, Barcelona Spain; ^21^ Department of Clinical Sciences, Clinical Memory Research Unit, Lund University, Lund Spain

## Abstract

**Background:**

Recent studies indicate that soluble tau phosphorylated at threonine 231 (*p*‐tau_231_) rises most prominently in the earliest preclinical stages of Alzheimer’s disease (AD), preceding overt amyloid‐beta (Aβ) PET positivity. Elevated *p*‐tau_231_ levels may promote emerging Aβ deposition, but the biological mechanisms linking early tau phosphorylation to subsequent Aβ accumulation remain unclear. Microglial reactivity may play a key role in Aβ dynamics, given their involvement in both facilitating Aβ clearance (protective) and promoting Aβ buildup via neuroinflammation (detrimental).

**Method:**

We studied 187 cognitively unimpaired (CU) individuals from the ALFA+ cohort who underwent repeated Aβ‐PET imaging over an average of 3.4 (SD=0.55) years (Table 1). Using linear regression models and mediation analysis, we tested the associations between cerebrospinal fluid (CSF) *p*‐tau_231_ levels, longitudinal Aβ accumulation measured by PET (annual centiloid change), and CSF sTREM2 levels, a biomarker of TREM2‐mediated microglial reactivity. Models were adjusted for baseline centiloid levels, age, *APOE*‐ε4 status, and sex.

**Result:**

Higher CSF *p*‐tau_231_ levels were associated with faster Aβ‐PET accumulation (β [95% CI]: 0.98 [0.26–1.69], *p*<0.01). Elevated *p*‐tau_231_ was also linked to increased CSF sTREM2 (β [95% CI]: 0.18 [0.11–0.25], *p*<0.001). Notably, higher sTREM2 was associated with reduced rates of Aβ accumulation (β [95% CI]: ‐1.67 [‐2.83 to ‐0.51], *p*<0.01), attenuating the relationship between *p*‐tau_231_ and Aβ‐PET accumulation by 27% (Figure 1).

**Conclusion:**

These findings suggest that soluble tau phosphorylation at early disease stages may enhance TREM2‐mediated microglial reactivity, which may in turn be a protective mechanism against Aβ aggregation. This may suggest that elevated *p*‐tau_231_ in the preclinical phase may initiate a microglial response that slows fibrillar Aβ accumulation (e.g., via phagocytosis). This may later transition to detrimental inflammation as the disease progresses. Enhancing microglial function in preclinical AD might therefore be a promising therapeutic strategy to delay or prevent Aβ buildup.